# Baiting and Feeding Revisited: Modeling Factors Influencing Transmission of Tuberculosis Among Deer and to Cattle

**DOI:** 10.3389/fvets.2018.00306

**Published:** 2018-12-04

**Authors:** Melinda K. Cosgrove, Daniel J. O'Brien, David S. L. Ramsey

**Affiliations:** ^1^Wildlife Disease Laboratory, Michigan Department of Natural Resources, Lansing, MI, United States; ^2^Department of Environment, Land, Water and Planning, Arthur Rylah Institute for Environmental Research, Heidelberg, VIC, Australia

**Keywords:** baiting, cattle, feeding, management, *Odocoileus virginianus*, simulation model, tuberculosis, white-tailed deer

## Abstract

Although tuberculosis caused by *Mycobacterium bovis* (bTB) is endemic in white-tailed deer (*Odocoileus virginianus*) in northeastern Michigan, USA, baiting and feeding of deer continue despite a regulatory ban. Previous modeling suggests aggregation at bait sites slows the rates at which harvest and/or vaccination decrease bTB prevalence, prolongs time to eradication, and increases the likelihood that once eradicated, bTB will re-establish following an incursion. However, the extent to which specific factors such as food density, attractiveness to deer, and persistence on the landscape influence bTB transmission is unknown. We used an individual-based, spatially-explicit stochastic simulation model of bTB in deer and cattle to investigate effects of feed density, attractiveness, and spatial and temporal persistence on bTB prevalence in deer and the probability of breakdowns in adjacent cattle herds. Because hunter harvest remains key to controlling bTB in deer, and harvest rates are in long term decline, we modeled these feeding-associated factors at harvest rates prevailing both when the model was developed (2003–2007) and in 2018. Food placement at randomized locations vs. fixed sites had little effect on bTB prevalence in deer, whereas increasing the probability that deer move to food piles (attractiveness) had the greatest effect of factors studied on both prevalence and herd breakdowns. Reducing food pile density reduced prevalence, but decreased herd breakdowns only modestly. Consistent availability of food over longer periods of time, as would occur with supplemental winter feeding or persistent recreational feeding, increased both prevalence in deer and cattle herd breakdowns dramatically. Though perhaps implausible to the public, altering how bait and feed for deer are used can reduce cattle herd breakdowns. Baiting and feeding bans have contributed to declining bTB prevalence, but non-compliance and continued legal sales of feed impede eradication. Requiring hunters to move food piles is unlikely to mitigate effects on transmission and is not a useful management tool. Compared to baiting, winter supplemental feeding or extended recreational feeding is likely to magnify bTB transmission by prolonging temporal availability. Because attractiveness of feed is influenced both by type of feed and deer behavior, research to quantify factors influencing deer movement to food should be a priority.

## Introduction

Infectious disease management in a wildlife reservoir is a contentious issue, especially when changes in human behavior are necessary. While there may be general agreement that control measures are warranted, the specific actions adopted are often controversial. The elimination of human-provided supplemental food for wildlife is of notable debate. Although the use of supplemental food for wildlife is recognized as a mechanism for both inter- and intra-species disease transmission (1–7), disagreements regarding the potential benefits vs. consequences related to this practice are still prevalent. Nonetheless, zoonotic diseases can have serious ecological and economic impacts (5, 8) and the disease management strategies chosen influence the magnitude of the impacts. Advocates for the use of supplement food as bait (an attractant during legal hunting seasons) argue that baiting is necessary to increase and maintain harvest for disease control, yet evidence for this is lacking (9, 10). However, the use of bait increases deer concentrations around these sites, increasing contact rates and so the potential for disease transmission. Supplemental food used in winter to (theoretically) aid survival can have similar consequences (5–7).

In Michigan, baiting and feeding of white-tailed deer (*Odocoileus virginianus*) is recognized as one of the biggest risks for transmission of tuberculosis caused by *Mycobacterium bovis* (bTB) (4, 8, 11, 12). In the wake of extensive logging and the decimation of deer populations by market hunting that occurred in the 1800s, large tracts of land were bought into private ownership and “hunt clubs” were established, both to conserve what deer remained and to provide populations for sport hunting and sustainable harvest. In the 1920's deer numbers in the northern Lower Peninsula of Michigan rose dramatically, in part due to lower harvest pressure on privately owned lands effectively maintained as refuges, and winter starvation became common (13, 14). In an attempt to reduce starvation, supplemental feeding became prevalent, yet starvation continued as the biological carrying capacity of the marginal habitat had been exceeded (13). During this same time, bTB reactor rates in cattle across the state were relatively high, reaching 20–30% in some counties (15, 16). Although Michigan was later successful in lowering bTB reactor rates in cattle and was eventually declared bTB free in 1979, contact between infected cattle and concentrated numbers of deer had been occurring for decades. Large scale supplemental feeding increased in the 1980's as competition increased between private land owners to attract deer to their property. It is hypothesized that spillover of bTB from cattle to deer occurred sometime during the 1950s to 1960s (17, 18), although deer were not recognized as a maintenance host until the 1990's (11). By that time bTB had become self-sustaining in the free-ranging deer herd and persists to the present day (11, 19).

Although bTB is endemic in deer in northeast Lower Michigan, winter feeding and baiting of deer continue despite a regulatory ban, albeit at lower levels than historically practiced. The sale of bait and feed remains legal and widespread, even where its use is banned (8). The current economic value of these sales is thought to be substantial. In 1995, it was estimated that baiting and supplemental feeding generated $15 million for Michigan farmers (20), and predictably, bans are typically opposed by farmers gaining from the sale of crops otherwise unmarketable for human consumption. This presents a persistent struggle to eradicate a contagious disease in the face of exacerbating practices. Previous modeling, using estimated levels of current baiting practices, suggests aggregation at bait sites slows the rates at which harvest and/or vaccination decrease bTB prevalence, prolongs time to eradication, and increases the likelihood that once eradicated, bTB will re-establish following an incursion (21). However, not well known are the extent to which specific factors such as feed site density, attractiveness to deer, and persistence on the landscape influence bTB transmission among deer and between deer and cattle.

Both direct and indirect interactions between livestock and wildlife have been well documented as a source of disease transmission (22–26). Increased bTB reactor rates in livestock resulting from alterations in supplemental food use for deer could be associated as well. We used an existing spatially-explicit model of bTB in deer and cattle (27) to evaluate how altering supplemental food density, attractiveness, and temporal and spatial persistence impact bTB prevalence in deer and the rate of cattle herd breakdowns.

## Materials and Methods

### Study Area

**Figure 1:**
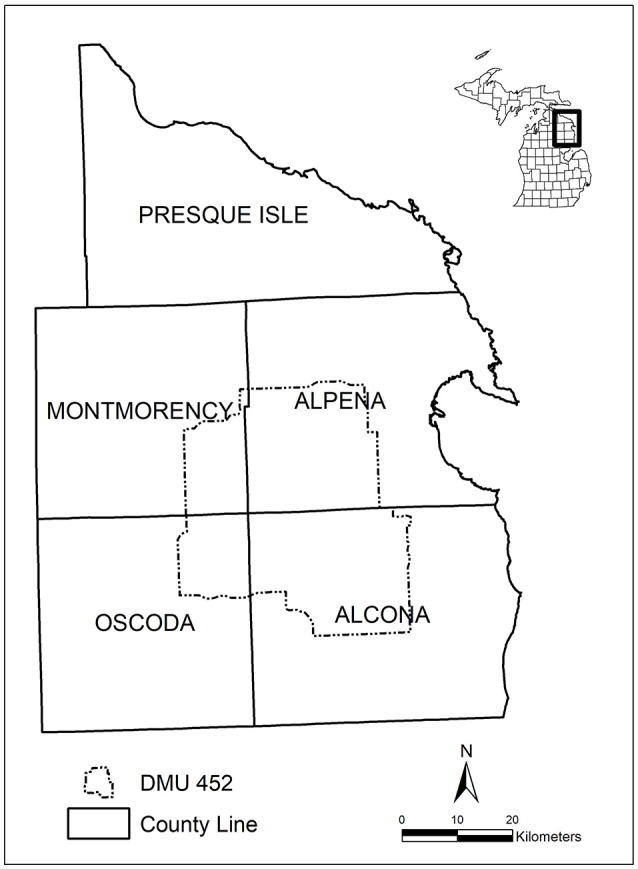
Simulation model study area, Deer Management Unit (DMU) 452, in northeastern Lower Peninsula of Michigan, USA.

We conducted simulations over a 48 x 51 km land area in the northeastern Lower Peninsula of Michigan consisting mainly of Deer Management Unit (DMU) 452 [12, 27, Figure 1). Deer Management Unit 452 includes parts of Alcona, Alpena, Montmorency, and Oscoda counties, is ~148,018 ha (1,480 km^2^), and comprised of 93% privately-owned land and 7% public land (28, 29). Topography, habitat, and deer management practices are described elsewhere (12, 30, 31). Historically, this area has defined the core outbreak area for bTB in Michigan (8).

### The Model

We used an individual-based spatially-explicit stochastic simulation model of bTB in white-tailed deer and cattle for all simulations. The structure, parameters, testing, validation, and assumptions of the model are described at length elsewhere (12, 27, 31). We used two Geographic Information System (GIS) layers to distribute deer and cattle across the landscape and account for movements and spatial concomitance that facilitate bTB transmission among deer and from deer to cattle. We used a deer layer that quantifies winter habitat potential for deer (as a surrogate for biological carrying capacity) to spatially distribute deer throughout the landscape (12, 31–33). We added a second layer that incorporated cattle producer locations, pasture areas, and cattle densities for all farms within and directly adjacent to DMU 452 to approximate cattle distribution (27). The model was originally calibrated to closely approximate steady-state age and sex specific bTB prevalence matched to long-term bTB surveillance data for deer from 2003–2007 (12, 31) and cattle herd breakdown rates 2003–2012 (27). A cattle herd breakdown is defined as a herd having at least one bTB reactor in the herd during whole herd testing.

Because rates of hunter harvest have declined over the last two decades, primarily due to demographic factors (34), the model was recalibrated in 2018 to adjust bTB transmission rates to accommodate current harvest rates. Briefly, sex- and age-specific harvest rates for DMU 452 were estimated from MDNR deer hunter harvest survey data via the sex-age kill method (35). Using those harvest rates, simulations were run to calibrate the sex-specific transmission rate parameters (betas) so that the predicted sex-specific bTB prevalence closely approximated field prevalence rates recorded from 2012–2016.

The baseline model simulates aggregation of deer around supplemental food sources by estimating the movement of a deer to the food source based on the location of the food source within its home range (12, 31). During each time step (2 months), each deer conducts a search of habitat cells within its home range. If a food pile is encountered, there is assumed to be a 0.2 probability of a change in the deer's current location to the food pile if it occurs at the center of its home range. That probability declines as a half normal function of the distance of the food pile from the home range center and was zero for food piles outside the home range. Food piles were randomly distributed across the landscape. Supplemental food was available from September to December, coinciding with deer hunting seasons in Michigan when bait is used.

In this study, we define supplemental food in two ways: (1) Baiting—the autumnal use of food to attract deer in an attempt to aid harvest; and (2) Feeding—the use of food for deer outside of legal hunting seasons (e.g., to facilitate wildlife viewing, or in an effort to aid winter survival). Our previous modeling suggests that aggregation of deer at food sites has a substantial effect on bTB transmission (12). However, that work only investigated the effects of the presence or absence of bait. The spatial nature of the model affords the ability to assess a variety of parameters associated with baiting and feeding. We altered four different supplemental food parameters to evaluate the effect on bTB prevalence in deer and cattle herd breakdowns over a 30-year period. Other model parameters were kept at default values as described previously (12, 27, 31). We ran each scenario under the original and current harvest rates for 5,000 replicates, discarding simulations for the first 50 and 150 years, respectively, (burn-in period). Due to the stochastic nature of the model, the burn-in period was required to ensure bTB and cattle breakdown rates had stabilized prior to changing the parameters under investigation. For comparison to our treatments, we conducted baseline simulations for both the original and current age- and sex-specific bTB transmission and harvest rates.

### Simulated Scenarios

#### Food Pile Density

Baiting and feeding is illegal in DMU 452 and has been since 1999 with limited use allowed in 2001 (8). However, a non-compliance rate of ~25% was estimated for hunters in the area (36). By dividing the number of potential baiters (determined from deer hunting license sales; see 12) by the area of DMU 452, the bait pile density was set at 0.02/ha. We evaluated the effect of reducing the non-compliance rate by 50% (e.g., via more stringent enforcement) which in turn reduced the bait pile density to 0.01/ha.

#### Food Pile Attractiveness

We define bait attractiveness as the probability that a deer will visit a bait pile if the bait pile is located within the center of the deer's home range. Attractiveness is influenced by both feed type and deer behavior and is thus difficult to quantify with certainty. Consequently, we evaluated three arbitrary variations of this probability: 0.05, 0.1, and 0.5, which were considered to be plausible bounds on the likely attractiveness of bait to deer, assuming the odds of deer being attracted to accessible bait are 50:50 or lower.

#### Spatial Persistence

Most hunters have preferred hunting locations and often establish permanent tree stands or deer blinds at these locations from which they hunt each year. In turn, if the hunter uses bait, the food piles are located in approximately the same location every year. We simulated the effect of requiring hunters to move food piles to new locations each year by randomizing pile locations, thus potentially affecting contact rates between habituated deer.

#### Temporal Persistence

Supplemental feeding commonly takes place during winter months when the public attempts to supplement reduced natural food sources for wild deer. This type of feeding can potentially aid winter survival, thus increasing deer densities to a level that exceeds the biological carrying capacity of the habitat. Both supplemental and prolonged recreational feeding (typically for the purposes of viewing) can unnaturally congregate deer for extended periods and in turn increase the probability of disease transmission. We evaluated the effects of prolonged food provision by humans on bTB prevalence and cattle herd breakdowns by expanding the temporal duration of food piles for two different time frames: September–February and September–April. The former simulates feeding through the most severe months of winter in Michigan, and the latter supplementing food through the entire winter.

#### Analysis

We used R (version 3.0.2, R Foundation for Statistical Computing, Vienna, Austria) for analyzing model output. Summary plots were generated for deer (bTB prevalence) over the simulated 30-year time frame. We compared output from the baseline simulation (no change to current deer management) to output from the simulations evaluating changes in baiting and feeding practices, to detect the direction and magnitude of influence on prevalence and herd breakdowns. Changes in bTB prevalence are expressed as absolute differences in year 30 vs. year zero of each simulation.

## Results

**Figure 2:**
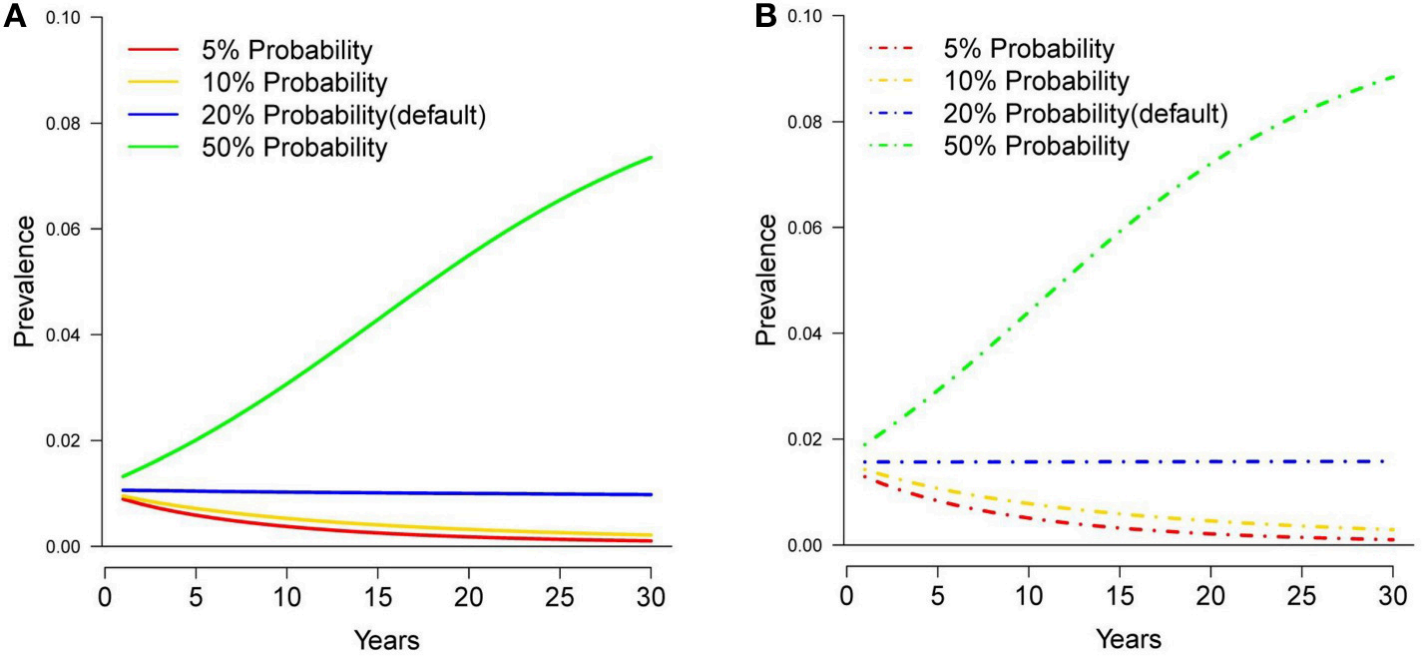
Tuberculosis prevalence in white-tailed deer over 30 years under different probabilities of deer movement to a supplemental food pile, assuming original **(A)** and current **(B)** harvest rates.

**Figure 3:**
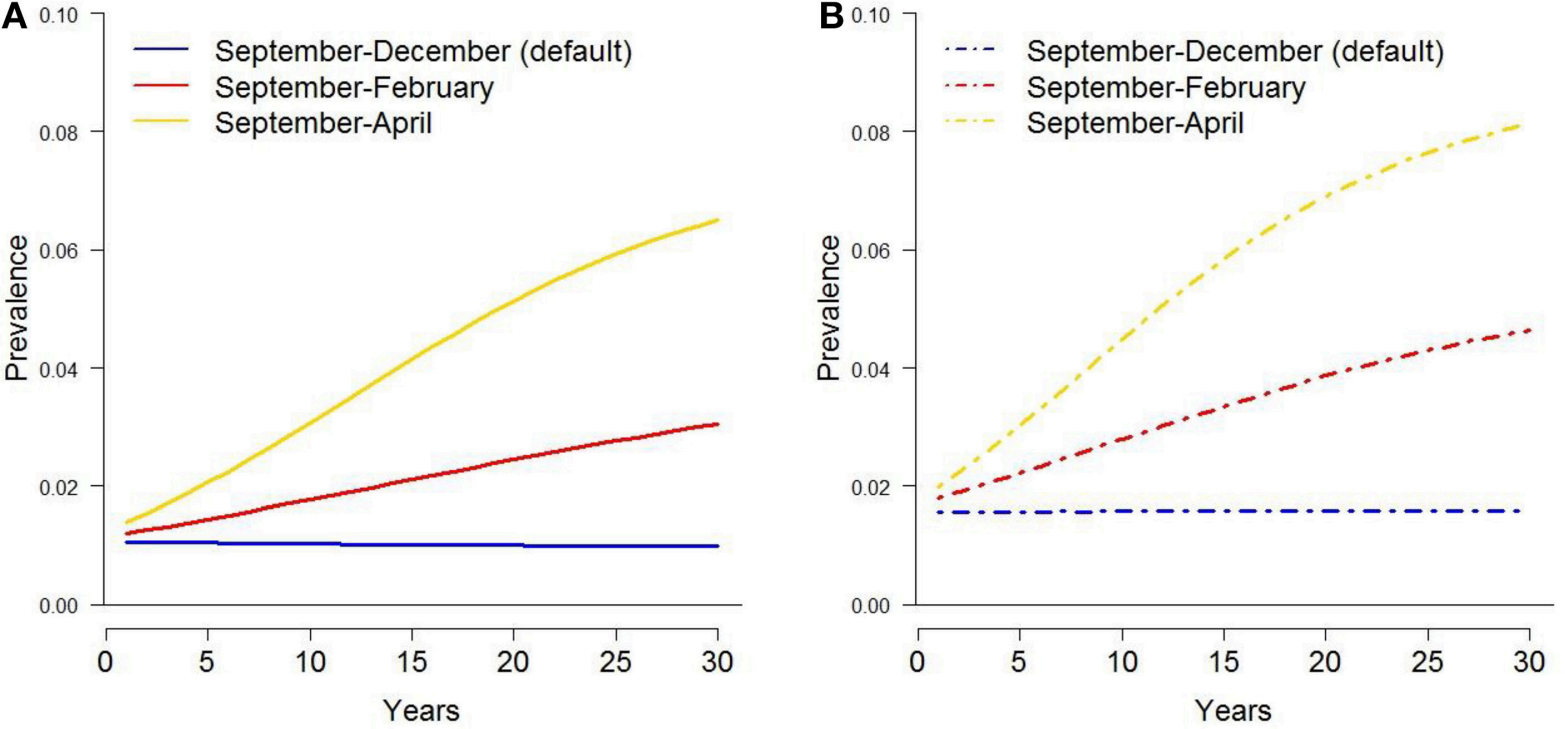
Tuberculosis prevalence in white-tailed deer over 30 years under extended temporal availability of supplemental food piles, assuming original **(A)** and current **(B)** harvest rates.

The baseline simulation for the original harvest rates resulted in a 0.001 decrease in deer prevalence after 30 years, and no change in prevalence over 30 years under the current harvest rates (Figures 2, 3). Cattle herd break downs were 2.8 per year on average under the original harvest rates and 3.4 per year under the current rates for the baseline simulations after 30 years (Table 1).

**Table 1:** Average number of cattle herd breakdowns per year under the original and current deer harvest rates after 30 simulated years.

	**Harvest rates**	
Scenario*	**Original**	**Current**
Baseline	2.8	3.4
Density 0.1	1.5	1.9
Prob 0.05	0.6	0.2
Prob 0.1	0.6	0.6
Prob 0.5	22.4	18.9
Random	2.5	3.0
Sept-Feb	9.3	9.8
Sept-Apr	20.2	17.9

* *Scenarios: Density 0.1 = Food Pile Density (0.01 food piles/ha); Prob 0.05, 0.1, 0.5 = Food Attractiveness (probabilities of deer moving to a food pile); Random = Spatial Persistence (randomizing food pile locations annually); Sept-Feb, Sept-Apr = Temporal Persistence (extension of time food piles are available)*.

### Food Pile Density

Reducing the baiting non-compliance rate (and thus food pile density) by 50% reduced bTB prevalence in deer by ~0.005 under both harvest rates, and reduced cattle herd breakdowns by an average of ~1.5 per year under both harvest rates (Table 1).

### Food Pile Attractiveness

Under the original harvest rates, reducing the probability that a deer visited a bait pile within its home range to 0.05 and 0.1, reduced prevalence by ~0.007 and 0.008 respectively, and by ~0.012 and 0.011 under the current harvest rates (Figure 2). Increasing the probability of visitation to a bait pile to 0.5 increased prevalence by 0.06 and 0.069 for the original and current harvest rates respectively. Cattle herd breakdowns were reduced to ~1 breakdown every 2 years under the original harvest rates and ~1 breakdown every 5 years under the current harvest rates for the visitation probability of 0.05. For the visitation probability of 0.1, breakdowns were reduced to ~1 breakdown every 2 years under both harvest rates (Table 1). However, increasing the probability of visitation to 0.5, increased herd breakdowns dramatically, ~22 per year under original harvest and ~19 per year under current harvest rates.

### Spatial Persistence

Changes in harvest rates notwithstanding, randomizing the location of bait piles each year had a negligible effect on bTB prevalence (0.002 decrease) in deer and on cattle herd breakdowns (Table 1).

### Temporal Persistence

Extending the time supplemental food was available by 2 months (i.e., through February) increased bTB prevalence by ~0.019 and 0.028 for the original and current harvest rates, respectively (Figure 3). When food was available through April, prevalence increased by ~0.051 and 0.061, respectively. Cattle herd breakdowns were approximately triple for both harvest rates after a 2-month increase in supplemental food (Table 1). Breakdowns were 5–7 times higher after a 4-month extension of supplemental food.

## Discussion

Over the 10 years since our model was first developed, there has been a decreasing trend in deer harvest, requiring the recalibration of our model. We simulated our scenarios under both old and new harvest rates, to illustrate the effect of this harvest reduction, and to enable comparisons to our previously reported findings. Many of the scenarios simulated under the current harvest rates showed increased bTB impacts as compared to the original harvest rates, emphasizing that hunter harvest remains an important factor controlling bTB in deer. Yet, hunter retention and recruitment are recognized as being in critical decline, and this may have detrimental effects for wildlife disease management (37–40). For density dependent diseases, such as bTB in deer, population control of wildlife reservoirs is crucial. However, after more than 20 years of providing increased harvest opportunity in DMU 452, harvest has likely been saturated and further extending harvest opportunity is unlikely to increase harvest (12). A survey of hunters and livestock producers in northeast Lower Michigan more than a decade ago indicated that only 23% of resident hunters supported a further reduction in deer numbers, whereas and somewhat surprisingly, only 57% of livestock producers in the area were in support of further reductions (41). Future bTB management in Michigan must take into consideration what can realistically be accomplished given the likelihood that reductions in harvest experienced over the past two decades are permanent. Thus far, policymakers have been reluctant to entertain other potential options for bTB control such as culling or vaccination, likely because prevalence in the deer population could be kept low via hunter harvest alone. However, as demographic changes continue to reduce the number of deer hunters (34), harvest may no longer be as efficacious as in the past.

In addition to deer density reduction, prohibiting the use of bait and feed for deer is generally one of the first control strategies implemented to limit disease transmission (5). However, a complete ban, in practice, is usually unattainable. Baiting and feeding bans have contributed to declining bTB prevalence in Michigan, but non-compliance, problematic prosecution of violators, and continued legal sales of feed impede eradication. Consequently, attempting to keep the non-compliance rate as low as possible becomes the goal. Even if a 50% reduction of the estimated non-compliance rate in DMU 452 could be achieved, our results show that only modest decreases in bTB prevalence and herd breakdowns would result. While we did not examine the effect of an increased non-compliance rate and an increase in food density, a resurgence in baiting may be occurring in DMU 452. Observations by MDNR field staff have noted an increase in supplemental feed sales (pers. comm. B. Mastenbrook). Moreover, it is not clear that a decreasing number of hunters will necessarily result in fewer bait sites on the landscape. If younger hunters are more likely to employ bait than the older hunters who are gradually leaving the hunting population, it is conceivable that the amount of bait on the landscape may not mirror the decline in hunter numbers.

If a complete prohibition of bait could be achieved, the benefits realized could be diminished by reduced hunter participation (42). In Michigan, there is a cultural significance to deer hunting, and traditions of deer hunting in some regions of the state have long included the use of bait (8, 42). Because reducing deer density is currently the primary strategy for bTB control, the willingness of hunters to harvest deer in endemic areas is crucial for success. If hunters are reluctant to hunt without bait due to the perceived increase in hunter effort, or if hunters choose to hunt elsewhere, baiting bans could potentially decrease harvest further. Following the 2001 hunting season in Michigan, a segment of hunters indicated through surveys that they eliminated or decreased their hunting activities due to the baiting ban (10, 42–44). However, participation and antlerless harvest were similar between DMU 452 and the remainder of the state where baiting remained legal (42). Notably, evidence suggests that in general, baiting does not increase harvest (9, 10, 20, 45).

Even if minimal baiting was allowed in an attempt to maintain hunter satisfaction, a method to mitigate the increased disease transmission resulting from supplemental food has not yet been found (6, 7). We simulated requiring hunters to move bait piles to new locations each year, but the practice had a negligible effect on bTB prevalence and herd breakdowns and is thus unlikely to be a useful management tool. Previous research has shown deer in northeast Lower Michigan to have a high fidelity to baited areas, but not to specific bait locations (46). Effectiveness notwithstanding, requiring hunters to move their bait sites is likely to be impractical. In DMU 452, thousands of hunters are spread over a 1,480 km^2^ area, >90% of which is privately-owned land. The effort necessary to enforce such an approach is not feasible and would arguably be more constructively applied to enforcing a strict baiting ban.

The more attractive a bait pile, the more likely a deer is to move to it. In our simulations attractiveness had a considerable impact on bTB prevalence and cattle herd breakdowns. Reducing the likelihood of a deer moving to a bait pile had a desirable impact on prevalence. However, increasing the probability of visitation to a bait pile resulted in 3.5-fold increase in prevalence and an average of 19 cattle herd breakdowns per year after 30 years. Hunters deliberately use bait piles to attract deer. Thus, expecting hunters to use less “attractive” bait defeats the purpose of using bait in the first place. However, attractiveness is also driven by deer behavior. Although supplemental food influences deer behavior (6, 46), quantifying that influence is difficult. Our modeling results are sensitive to variation in this parameter [see (12), Appendix A) and research to better quantify the combined effect of food attractiveness and deer behavior would be valuable.

Compared to baiting, winter supplemental feeding or extended recreational feeding is likely to magnify bTB transmission by prolonging temporal availability. In our simulations, each 2-month extension of food availability increased bTB prevalence 2–3% and herd breakdowns increased dramatically (Table 1). The longer food sites are maintained, the greater the cumulative transmission of disease over time (6, 7, 47). These simulations suggest that even 5 years of feeding throughout the winter increased prevalence by more than 50%. Although historic large-scale winter feeding (11) has decreased significantly, a resurgence of this practice could wipe out gains made in bTB control to date after only a few years. Should managers ever allow winter feeding to become widespread again, knowing the likely consequences, the temporal window during which it is allowed must be a critical consideration.

Our temporal persistence scenarios extended the time bait was available on the landscape to simulate winter feeding, a reasonable but imperfect approximation. Bait is used theoretically to aid harvest in autumn. Winter feed sites occur without harvest and generally contain a greater quantity of food than bait sites, potentially attracting more deer to the site. Yet there are usually fewer feed sites in winter than bait sites during the hunting seasons. Historic winter feed site locations were documented previously via aerial surveys when winter feeding was extensive. Future research could incorporate these sites into our model as a new GIS layer to more precisely estimate site densities for comparison against these results.

**Figure 4:**
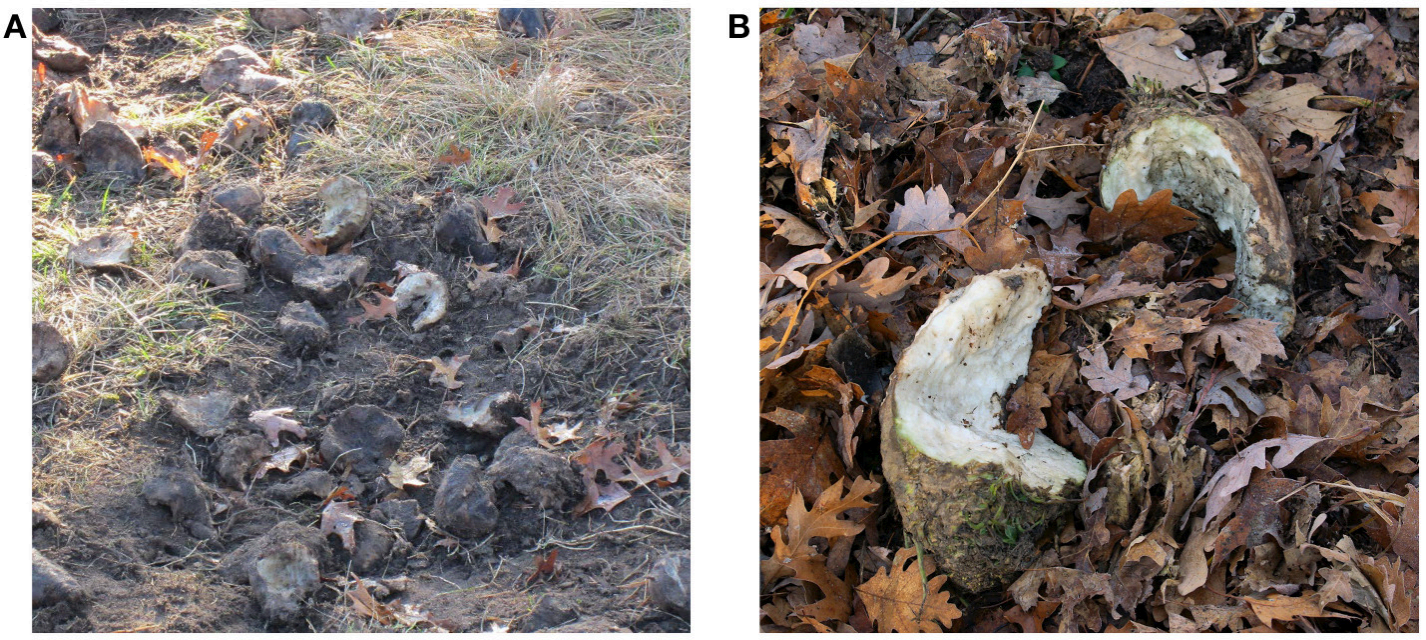
Sugar beets (*Beta vulgaris* subsp. *vulgaris* convar. *vulgaris* var. *altissima*) in an illegal deer baiting site, Alcona County, Michigan. **(A)** Hoof traffic exposes soil which may act as a source of indirect exposure to *M. bovis* when contaminated by infected deer. **(B)** Teeth marks on sugar beets partially eaten by deer. *M. bovis* likely survives for extended periods on these foodstuffs under the cold, wet conditions typical of autumn and winter in northeastern Michigan.

Our model does not explicitly account for indirect transmission of bTB resulting from environmental contamination; if it did, we hypothesize that both prevalence of bTB in deer and the number of cattle herd breakdowns would likely increase, although the magnitude of those increases is uncertain. Deer infected with *M. bovis* shed infectious bacteria in oronasal secretions (48), and food items contaminated by infected deer are infectious for susceptible deer (49). In a field setting, bait and supplemental feed sites facilitate both the contamination of feedstuffs and surrounding soil with saliva and nasal secretions of deer feeding there (Figure 4), and so act as an efficient source of bTB transmission even after both the infected deer, and perhaps the feed itself, are gone. Experimental studies conducted both under laboratory (50) and Michigan outdoor (51, 52) conditions have shown that *M. bovis* can remain viable on feedstuffs for days to several months, depending on the substrate and ambient conditions such as temperature, humidity, and shade. That said, efforts to isolate the bacteria from documented deer feeding sites in Michigan have thus far proven unsuccessful (53). Although yet to be explicitly shown in Michigan field settings, research from other bTB-infected ecosystems has elegantly demonstrated the efficacy of environmental substrates such as watering holes as sources of bTB exposure (54). To parameterize indirect environmental transmission in our model, further research to determine the visitation rates of deer to sites where food was once, but is no longer, present would be necessary, along with rates of soil ingestion. Persistence times for *M. bovis* could be drawn from existing distributions of bacterial survival on foodstuffs (50). Alternatively, a value for indirect transmission could be estimated arbitrarily, and subjected to sensitivity analysis as in previous work (12). In any case, our model has demonstrated the importance of bait piles for bTB transmission in WTD, notwithstanding the additional risk due to environmental contamination.

No matter how feed sites are used, their presence, and potentially their past presence, increases bTB prevalence (12). Aggregation, crowding, increased competition, exposure to unrelated individuals, and increased predator-prey interactions are a few of the consequences of feed sites (5–7, 46, 55). These effects in turn increase stress and lower immune response, increasing susceptibility to disease (7, 56). Supplemental feed is often of lower quality than naturally available food and does not provide complete nutrition (57), leading to deficiencies and lowered immune response. Feeding deer to aid winter survival can be successful if begun early, but resulting increased survival and fecundity can increase densities, increasing bTB transmission. Feeding later in winter after nutritional deficits are realized often does not aid survival because body condition is often too poor to be reversed, putting these deer at even greater disease risk (57).

Our results indicate that altering how bait and feed for deer are used can reduce, or increase, cattle herd breakdowns, which is frequently implausible to the public. Discussions regarding the use of bait and feed are often considered of relevance only to bTB transmission among deer. While not effective as the sole management tool (27), how food provided by humans for deer impacts broader issues of bTB eradication from cattle must be considered by both regulators and agricultural producers. While deer bait and feed provide a market for crops that have limited marketability as human food, agricultural stakeholder groups should carefully consider the trade-offs between income generated for crop farmers vs. the economic costs of herd breakdowns to the cattle industry and the larger agricultural economy. Such introspection has largely been lacking in Michigan thus far.

Managing disease in deer must not preclude the use of other means of disease management for livestock. Increased biosecurity on farms in areas where bTB is endemic should remain a priority. Brook et al. (25) argue that a “bottom-up” approach for reducing transmission risk at the wildlife-livestock interface would be more practical and effective. This approach tailors risk mitigation to the individual farm level, taking into consideration spatial overlap and resources, winter feeding areas, animal behavior, fencing, and farm management, in contrast to relying primarily on wildlife culling or harvest, disease testing, baiting and feeding regulations, and cattle depopulation. Clearly, changing farm management can reduce disease transmission between wildlife and livestock substantially (23, 25, 26, 58–60). Our model includes a parameter to account for the proportional reduction in deer-to-cattle contact likely to be afforded by increased biosecurity [see Equation 1 in (27)]. For this study, we chose to hold that parameter at its default value of 1 (unmitigated deer to cattle contact). While heightened biosecurity could help reduce the effects of increased deer baiting and feeding, previous work suggests quite a high level of biosecurity would be necessary in order to have a high probability of reducing herd breakdowns [Figure 10 in (27)]. Although increased biosecurity alone is unlikely to eliminate herd breakdowns in the absence of broader measures to control bTB in deer (27), improving farm management in conjunction with altering supplemental food use for wildlife may facilitate reductions in interspecies bTB transmission.

## Conclusion

The use of supplemental food for deer continues to be one of the biggest regulatory challenges to bTB eradication in Michigan. As long as widespread baiting and feeding continue, successful eradication of bTB is likely unattainable. As wildlife managers learn to compensate for the decline in hunter numbers and adjust to changes in hunter demographics, the challenges for disease management become more complex. Even if a low level of bTB in deer is acceptable to the public, the ever present and serious risks to livestock remain problematic. Our modeling results demonstrate that a link between supplemental feeding of deer and occurrence of bTB in livestock exists and that feeding has implications not only for deer. Wildlife management necessarily involves managing people and their behaviors as well as wildlife populations. Disease management programs need to include educating people on how perceived short-term benefits from the use of bait and feed can lead to adverse long-term consequences. Convincing people not only to change their own behavior, but to also encourage others to do so, requires a culture change. Invoking this change is one of the most difficult challenges wildlife disease managers face.

## Author Contributions

DR developed and recalibrated the model. DO provided the project concept and design. MC conducted the simulations, analyzed output, and drafted the manuscript. All authors contributed to intellectual material, manuscript review, and editing, and have approved the submitted manuscript.

### Conflict of Interest Statement

The authors declare that the research was conducted in the absence of any commercial or financial relationships that could be construed as a potential conflict of interest.
